# Use of Bacterial Cellulose and Crosslinked Cellulose Nanofibers Membranes for Removal of Oil from Oil-in-Water Emulsions

**DOI:** 10.3390/polym9090388

**Published:** 2017-08-23

**Authors:** Enas Hassan, Mohammad Hassan, Ragab Abou-zeid, Linn Berglund, Kristiina Oksman

**Affiliations:** 1 Cellulose and Paper Department & Centre of Excellence for Advanced Sciences, National Research Centre, 33 El-Behouth Street, Dokki 12622, Egypt; ea.hassan@nrc.sci.eg (E.H.); re.abouzeid@nrc.sci.eg (R.A.); 2 Egypt Nanotechnology Centre, Cairo University, El-Sheikh Zayed, 6th of October City 12588, Egypt; 3 Department of Engineering Sciences and Mathematics, Luleä University of Technology, Luleä SE 97187, Sweden; linn.berglund@ltu.se (L.B.); kristiina.oksman@ltu.se (K.O.)

**Keywords:** bacterial cellulose, palm fruit stalks, cellulose nanofibers, oil emulsion, membranes

## Abstract

Never-dried bacterial cellulose (BC) and crosslinked cellulose nanofibers (CNF) were used for the removal of oil from stabilized and non-stabilized oil-in-water emulsions with droplet sizes less than 1 µm. The CNF membranes were exchanged with isopropyl alcohol before drying. The microscopic structure of the prepared membranes was evaluated using scanning electron microscopy (SEM); the water flux and the rejection of oil were evaluated using a dead-end filtration cell. BC harvested after different incubation time periods (2 to 10 days) did not show a change in the width of the nanofibers, but only the thickness of the membranes was increased. Pure water flux was not affected as a result of increasing thicknesses of BC membranes harvested after 4–10 days while BC harvested after two days had significantly higher water flux than the others. BC showed a higher flux and efficiency in removing oil from oil emulsions than CNF membranes. Removal of oil by the different membranes from the non-stabilized oil emulsion was more efficient than from the stabilized one.

## 1. Introduction

Use of membrane technologies, such as microfiltration, ultrafiltration and nanofiltration, for water purification and treatment are continuously increasing for providing clean water. Most commercially-available membranes are produced from synthetic polymers from fossil resources [1]. Production of these membranes usually requires large quantities of solvents and chemicals. There is increasing interest to produce membranes based on natural polymers, especially those based on nanocellulose, such as cellulose nanofibers and bacterial cellulose. Cellulose nanofibers (CNF) can be isolated from cellulose fibers of wood or agricultural residues using different technologies, such as grinders, high-pressure homogenizers, or ultrasonicators [2,3]. Cellulose nanofibers can be also prepared using electrospinning technology via dissolution of cellulose or its derivatives in suitable solvents, followed by spinning under high electric voltage [4]. Most recently, cellulose nanofibers were prepared using forcespinning technology where limitations of electrospinning, such as using high-voltage and low production rate, are avoided since centrifugal force, rather than electrostactic force, is used for spinning [5,6].

Cellulose nanofibers can be shaped into nanoporous membranes without the need to use solvents and casting as in phase inversion technique. Using cellulose nanofibers as a naturally-occurring and renewable material in ultrafiltration membranes, especially their use in making porous ultra-thin films, recently attracted increasing attention [7].

On the other hand, bacterial cellulose (BC) is a pure form of cellulose that can be synthesized by some microorganisms, such as *Acetobacter xylinum* and *Gluconacetobacter hansenii* bacteria, under static or dynamic cultures [8,9,10,11]. The produced BC is highly porous with a network structure and small pore size, which qualify it for use in filtration purposes. However, research on using BC as ultrafiltration membranes has not been widely investigated; relatively few studies were published on using non-modified and modified BC as membranes for water treatment. For example, BC areogel was surface-modified by trimethyl silane and used for removal of oil and some organic solvents from water [12]. A BC membrane with a thickness less than 6 µm harvested after two days of incubation was tested as a membrane for filtration of *Chlorella* sp. and bovine serum albumin [11]. The studied BC membranes showed rejection efficiency, e.g., the capability of removing the targeted materials, of about 99%. BC/graphene oxide composite membrane was prepared by dispersing graphene oxide in BC formamide gel [13]. The prepared membranes are expected to have selective ion permeation properties and potential applications in the separation field. Nanopaper sheets prepared from BC were tested as ultrafiltration membranes for the removal of polyethylene glycol (*M*. *wt* 93 kDa) with a rejection efficiency of 75% [14]. Laminated composites from BC and β-chitin or deacetylated chitin sulfonate were prepared and tested for removal of polyethylene glycol (*M. wt* 50 kD); rejection values of 85–90% were obtained as compared with 30% rejection of neat BC membrane [15].

Removal of oil from oily wastewater produced from different industries, such as food, petrochemicals and pharmaceutical industries, is mandatory due to environmental concerns and to save our water resources [16]. Ultrafiltration membranes, from both natural and synthetic polymers, were used for the removal of oil from oil-in-water emulsions. Regenerated cellulose and cellulose derivatives were studied for making membranes to remove different types of oils from oil-in-water emulsions [17,18,19,20]. Recently, cellulose nanofibers were used as ultra-thin films membranes for the same purpose [21]. In that work, 2,2,6,6-tetramethylpiperidine-1-oxyl (TEMPO)-oxidized CNF ultra-thin membrane with thickness of about 0.1 µm formed over polyacrylonitrile/polyethylene terphthalate (PAN/PET) support was used for oil removal from stabilized oil-in-water emulsion. At a pressure of 207 kPa and after 48 h, flux of the emulsion was 208 L/h/m^2^ while the rejection efficiency was >99%; the test was carried out at 40 ± 2 °C.

However, to the best of our knowledge, use of membranes made from BC for the removal of oil from oil-in-water emulsions has not been studied so far. In addition, the use of BC in its never-dried state as a membrane has not been studied either. The aim of the current work was to study the use of never-dried bacterial cellulose and crosslinked CNF as membranes for removal of oil from stabilized and non-stabilized oil emulsions.

## 2. Materials and Methods

### 2.1. Materials

Date palm fruit stalks were obtained from local fields in Giza, Egypt after separating dates from the stalks. Castor oil, sodium hypochlorite, sodium hydroxide, sodium chlorite, acetic acid, ethanol, isopropyl alcohol, glucose, peptone, yeast extract, Na_2_HPO_4_, citric acid, glucose, peptone, yeast extract and sodium bromide were reagent grade chemicals and used as received (Sigma-Aldrich, St. Louis, MO, USA). Polyamide-amine-epichlorohydrin (PAE) was commercial grade (solid content ~ 33 wt %, Solines, Wilmington, DE, USA). PAE solution was diluted to 1 wt % with distilled water before use. An anionic surfactant was used for preparation of the stabilized oil emulsion (Texapon^®^ P, Cognis, Monheim, Germany).

### 2.2. Preparation of Palm Fruit Stalks Pulp

Bleached pulp was prepared from the stalks as described before [22]. Date palm fruit stalks pulp was obtained by alkali treatment using 15% NaOH (based on oven-dried weight of the stalks) at 150 °C for 2 h. The unbleached pulp was bleached by sodium chlorite/acetic acid mixture at 80 °C for 1 h [23]. The composition of the bleached pulp was determined using the previously-published methods [24] and was: α-cellulose, 71.5%; pentosans, 18.4%; degree of polymerization (DP), 1264; and 0.64% ash.

### 2.3. Isolation of Cellulose Nanofibers(CNF)

Isolation of CNF from bleached pulp was carried out as previously described [25]. In brief, the bleached pulp suspension with 2% consistency was first disintegrated by high-shear mixer. Then the fibers were refined using a high-shear ultrafine friction grinder (MKCA6-2, Masuko Sanguo, Kawaguchi, Japan); a 9 µm gap between the disks was used and the fibers were run through the grinder for about 100 min.

### 2.4. Preparation of Bacterial Cellulose Membranes

The bacterial strain *Gluconacetobacter xylinum* was used for bacterial cellulose production. Bacterial stock cultures were maintained at 4 °C in 250 mL Erlenmeyer flask. Bacterial cellulose was produced in 250-mL Erlenmeyer flask containing 100 mL of Hestrin-Schramm liquid medium constituted of 5 g/L yeast extract, 20 g/L glucose, 5 g/L peptone, 1.15 g/L citric acid, 2.7 g/L Na_2_HPO_4_; the pH was adjusted to 6 at 28 °C for up to 10 days [11]. After incubation, the produced pellicles were harvested and washed with water to remove residual media. Pellicles were then treated with 0.1 M NaOH at 80 °C for 1 h and finally washed repeatedly with water until a neutral pH was obtained. The pellicles were kept wet in 0.1% sodium azide solution in the fridge at 5 °C until use. The diameter of the obtained wet pellicles was about 7 cm.

### 2.5. Characterization of CNF and BC

Transmission electron microscopy of CNF was carried out using high-resolution transmission electron microscopy, HR-TEM (JEM-2100 transmission electron microscope, JEOL, Tokyo, Japan). Scanning electron microscopy of BC was carried out using high-resolution scanning electron microscope (Zeiss Merlin FEG-SEM, Zeiss, Oberkochen, Germany) while scanning electron microscopy of CNF was carried out using an FEI Quanta 200 scanning electron microscope (FEI Company, Eindhoven, The Netherlands). Water in the wet BC pellicles was first exchanged with isopropyl alcohol then freeze-dried before SEM examination. Atomic force microscopy (AFM) of the isolated nanofibers was carried out using a Veeco MultiMode scanning probe microscope (Veeco Instruments Inc., Plainview, NY, USA) equipped with a Nanoscope V controller. A droplet of the aqueous fiber suspension was dried onto a mica surface prior to AFM examination and images were collected using a tapping mode etched silicon tip with a nominal spring constant of 5 N/m and a nominal frequency of 270 kHz.

### 2.6. Preparation of Oil Emulsions

Castor oil was used for the preparation of oil-in-water emulsions. One gram of oil was mixed with 1000 mL of distilled water without or with addition of the anionic surfactant (15% based on weight of oil) and homogenized using a Hielscher ultrasonic processor (Hielscher UP400s, Teltow, Germany) for 15 min in an ice bath. Particle size distribution of the obtained emulsion was measured using a zetasizer instrument (Malvern Instruments, Malvern Worcestershire, UK).

### 2.7. Cellulose Nanofiber Membrane

CNF (0.02 g oven-dry weight) in water suspension with concentration of 0.1 wt % and 4% PAE crosslinker (based on oven-dry weight of CNF) were filtered on 9-cm hardened filter paper using vacuum pump. The formed wet membrane was first exchanged by isopropyl alcohol, then dried at 105 °C for 30 min. The grammage of the formed CNF membrane was 2.5 g/m^2^.

### 2.8. Evaluation of Membrane Properties

#### 2.8.1. Pure Water Flux

The water flux was measured using a 300-mL dead end cell, (Sterlitech HP 4750, Sterlitech, Kent, WA, USA). Before the measurements, 5-cm diameter discs were cut out from the membranes and soaked in distilled water for one hour to ensure equilibration of the membrane. The conditioned membranes were placed on a stainless steel porous support disk in the dead end cell; water was passed through the membranes at a differential pressure of 1 MPa maintained using N_2_ gas at room temperature. The quantity of water that passed through the membrane was weighed accurately for a defined time interval; the flux was calculated (L/h/m^2^) for the active filtration area (14.6 cm^2^).

#### 2.8.2. Rejection Efficiency

The capability of CNF and BC membranes to remove oil from the oil-in-water emulsions was evaluated using a dead-end cell as mentioned above. The filtrate was collected and the turbidity of the filtrate was examined using UV-VIS spectrometer (Shimadzu, Tokyo, Japan). A standard curve was first estimated from the absorbance of oil emulsions with different concentrations, from 10 to 100 mg of oil per liter (Supplementary Information, Figure S1). The concentration of the residual oil in the filtrate was calculated from the equation of the standard curve. The rejection efficiency of the membranes to remove oil from water was calculated using the following formula:

Rejection(%) = ((Concentration of oil after filtration/Concentration of oil before filtration)) × 100
(1)

## 3. Results and Discussion

### 3.1. CNF and BC

**Figure 1:**
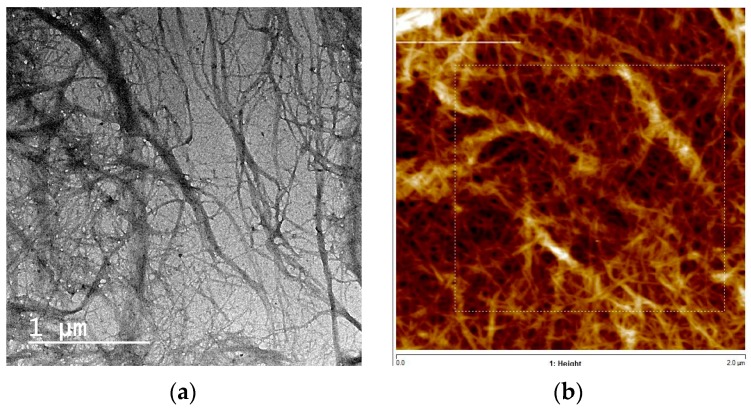

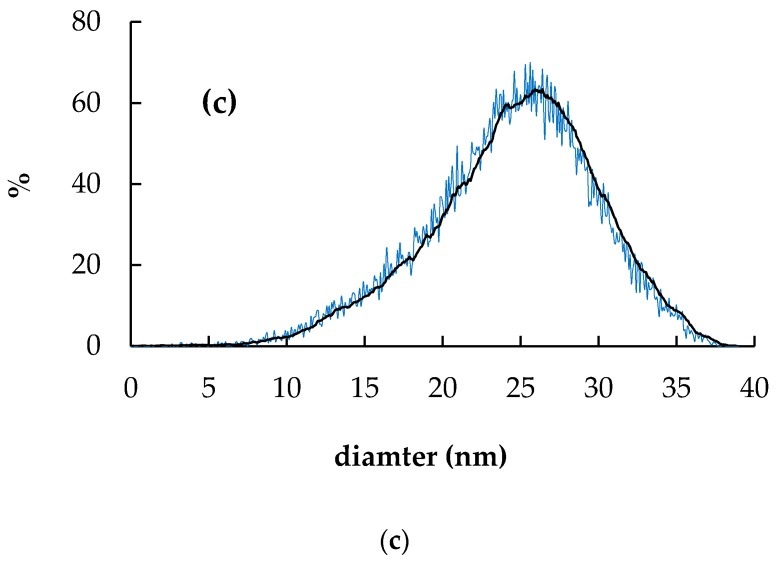
(**a**) TEM image of CNF isolated from bleached palm fruit stalks pulp, (**b**) AFM image of CNF and (**c**) diameter distribution curve of the CNF measured.

Palm fruit stalks are characterized by their high content of cellulose fibers that have similar fiber dimensions to many hardwood and agricultural residues [22]. The isolated nanofibers in the current work was characterized using HR-TEM and AFM (Figure 1). As the TEM image shows, palm fruit stalk CNF isolated using ultrafine grinding show a width in the range of about 13–25 nm; fibrils with larger widths of about 75–100 nm were also noticed. An AFM image of CNF based on the depth measurements show a range of diameters from about 10–35 nm.

**Figure 2:**
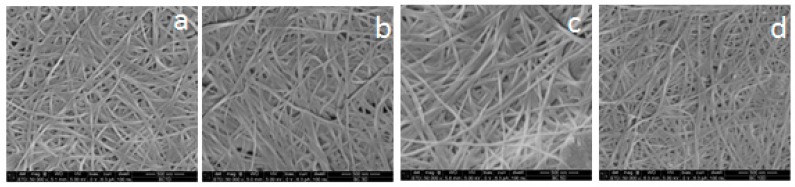
SEM images of freeze-dried BC harvested after (**a**) two days, (**b**) four days, (**c**) six days and (**d**) 10 days (magnification: 50,000×).

**Figure 3:**
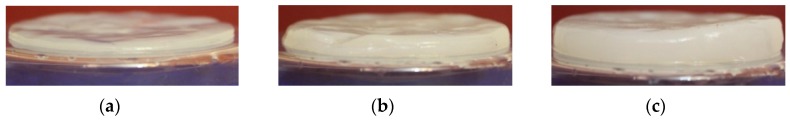
Photos show thickness of never-dried BC harvested after (**a**) 2 days, (**b**) 6 days and (**c**) 10 days.

On the other hand, BC showed a highly-porous structure with fibril diameters in a narrow range from about 25 to 45 nm (Figure 2). It was noticed that the width of BC nanofibers did not significantly change as the harvesting time increased, but the thickness of the obtained film increased due to greater mass of BC being formed (Figure 3); Table 1 shows the weight, thickness and water content of the prepared BC.

**Figure 4:**
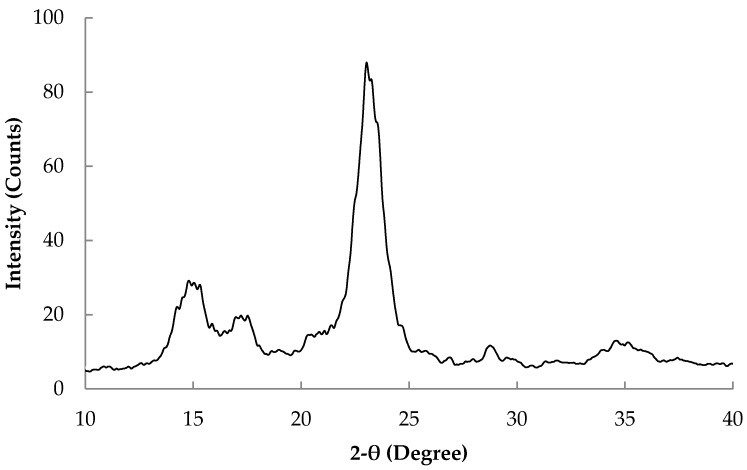
XRD pattern of BC harvested after 2 days.

The crystalline structure of the prepared BC was briefly tested for the sample harvested after two days and the X-ray diffraction (XRD) pattern is shown in Figure 4. The pattern obtained shows the cellulose I-β structure with main reflection peaks at 2-θ angles of 14.4, 16.7, 22.9 and 34.2 due to reflections from <1–10>, <110>, <200> and <004> planes, respectively [26]. Crystallinity calculated according to the following equation [27] from the intensities of the peak at 2-θ = 23 (*I*_200_) and the minimum at 2-θ= 18 (*I*_am_) was 92%, which is in accordance with previously published data of BC [28]: CrI = (*I*_200_ − *I*_am_)/*I*_200_, where *I*_200_ is the intensity of the diffraction at the position of 200 peak (2-θ = 22.7) and *I*_am_ is the intensity at about 2-θ = 18.

### 3.2. Castor Oil Emulsions

Castor oil emulsions are widely used in different applications such as food, pharmaceutical and industrial products, including lubricants, varnishes, printing inks and coatings. Among the different oils, castor oil is characterized by high purity, consisting of 90% ricinoleic fatty acid [29]. Therefore, emulsions with homogeneous particles size distribution are expected to be produced from castor oil.

**Figure 5:**
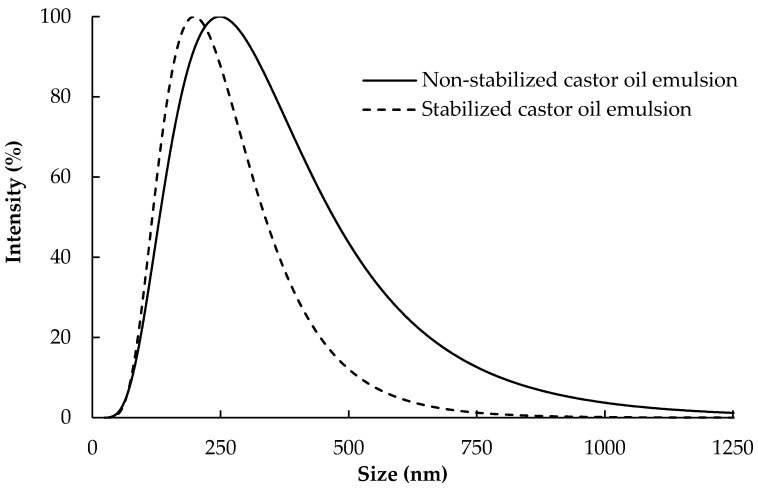
Gaussian distribution of diameter of stabilized and non-stabilized castor oil-in-water emulsions.

**Figure 6:**
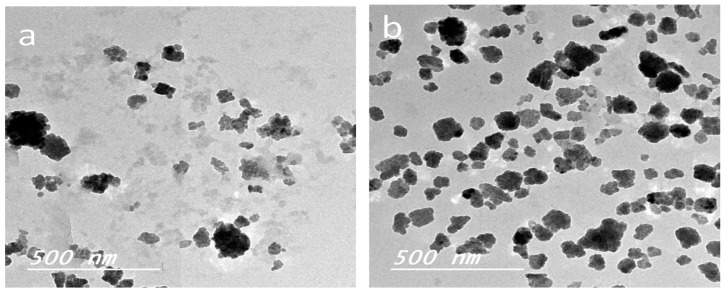
TEM images of castor oil-in-water emulsions: (**a**) non-stabilized and (**b**) stabilized.

Figure 5 shows the Gaussian distribution of oil droplet sizes as measured by the zetasizer instrument. The average diameter of the oil droplets in the case of non-stabilized and surfactant-stabilized oil emulsion was 376 ± 256 and 220 ± 99 nm, respectively. TEM images of the oil emulsion droplets (Figure 6) were in accordance with the particles size analysis obtained using the zetasizer measurements.

### 3.3. BC and CNF Membranes

**Figure 7:**
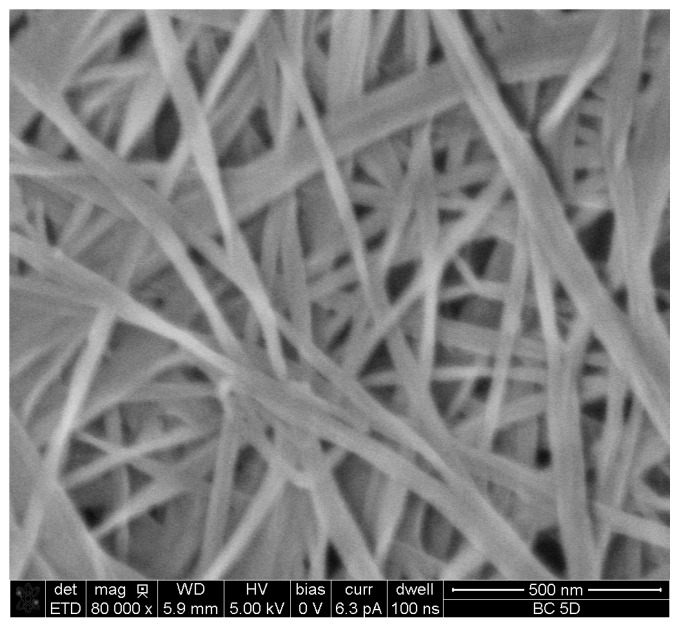
SEM image of BC harvested after 6 days (magnification 80,000×).

The produced BC pellicles were used as membrane without drying. As mentioned above in Table 1, the dry weight of the pellicles was small (0.017 g for the two-day BC to about 0.44 g for the 10-day BC), as well as the dry thickness. The non-woven structure of BC is unique in terms of porosity, compactness, tightness and wet strength. A highly-magnified SEM image (Figure 7) showed that the pores width at the surface of the BC harvested after 6 days is in the range from 20 to 85 nm. SEM images at the same magnification of the other BC membranes harvested at different times showed that the pores width at the surface was in the same range (Supplementary Information, Figure S2). Calculation of porosity from the dry and wet weight, thickness and diameter of BC pellicles according to the previously-published following equation [30] showed that the porosity of the different BC samples was in the range of about 95%–97%.

Porosity = (*W*_w_ − *W*_d_)/ (*d* × *A* × *D*) (2)

where *W*_w_ and *W*_d_ are the weight of wet and dry membranes, respectively, *d* is the density of water, *D* is the thickness of the membrane and *A* is the area of the membrane.

**Figure 8:**
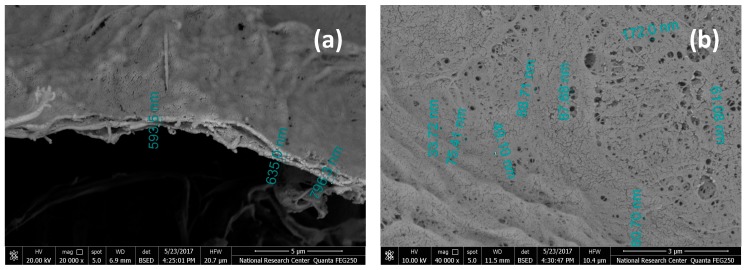
SEM images of: (**a**) cross section and (**b**) surface of alcohol-exchanged CNF films formed on a hardened filter.

In the case of CNF, a thin film of crosslinked CNF with a basis weight of about 2.5 g/m^2^ was formed on hardened filter paper. The average thickness of the thin film formed was about 0.7 µm (Figure 8). The images showed that the diameter of the pores at the surface of the CNF membrane ranged from about 61 to 172 nm. Measuring porosity of the CNF membrane using the wet and dry weight of the membrane was not possible due to the very small thickness of the CNF layer over the filter paper and also the difference in weight of the wet and dry membranes (filter paper + CNF) was not significant.

### 3.4. Water Flux of CNF and BC Films

Table 2 shows water flux values for filtration of about 300 mL of distilled water using CNF membrane and BC membranes harvested after different time intervals. BC membrane harvested after two days had exceptionally very high water flux (845 L/h/m^2^) because of its small weight and thickness compared to other membranes harvested at longer times. BC membranes harvested after 4, 6 and 10 days had average water flux values of about 448, 441 and 498 L/h/m^2^, respectively. These close flux values could be due to that the diameter of the nanofibers forming these BC membranes, pores size at the surface and porosity are very close to each other. It is known that the pores size of the membrane and thus the flux, is generally governed by the diameter of the fibers forming the membrane [8]; the smaller the diameter of fibers, the narrower the pores and, thus, the lower the flux, and vice versa. On the other hand, CNF membranes had much lower water flux than BC membranes although the former showed larger pore sizes at the surface and also much smaller thickness. This could be due to that BC membranes were never-dried and also due to the unique structure of the BC membrane. Drying CNF and their crosslinking will result in much more compact structures with lower hydrophilicity and swelling than the never-dried BC. In addition, hydrated polymers, such as never-dried BC, have little resistance to water flow due to high water-polymer interaction, while dried ones, such as CNF used in the current work, have less water-polymer interactions that, in turn, lowers the water flux across the membranes [31]. Additionally, the CNF membranes are expected to have relatively lower average free-volume pore size due to membrane shrinkage during drying as compared to the never-dried BC [32].

### 3.5. Flux of Oil Emulsions

**Figure 9:**
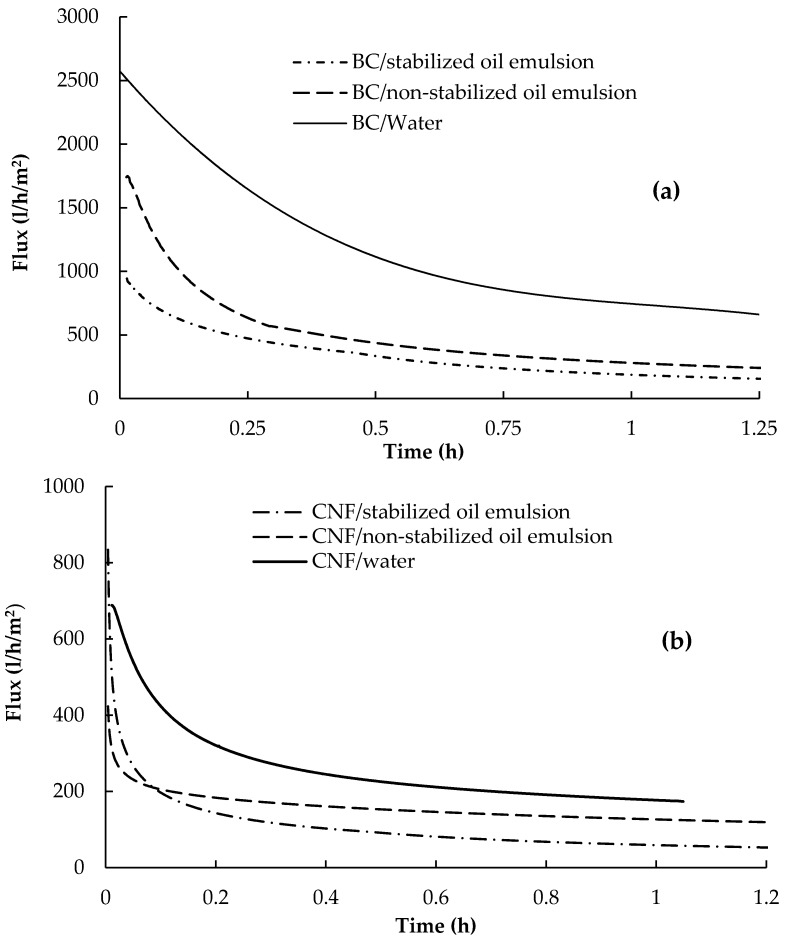
Flux curves of (**a**) bacterial cellulose and (**b**) CNF membranes for filtering stabilized and non-stabilized oil emulsions.

The flux of oil emulsions through CNF and BC membranes was tested and the flux versus time curves are presented in Figure 9. The results of using BC membranes harvested after two days are presented since they had the highest pure water flux, as seen above. Non-stabilized and stabilized emulsions with oil concentration of 100 mg/L were used. As can be seen from the curves, BC membranes hadmuch higher oil emulsions flux than CNF membrane. The flux of the non-stabilized oil emulsions was higher than the stabilized one due to the lower size of oil droplets in the case of the stabilized emulsion, which can cover larger area of the membrane and fill its pores. In addition, aggregation of non-stabilized oil droplets into larger ones is expected, which makes the filtration easier than in the case of the stabilized oil emulsion. Flux values of 261 and 136 L/h/m^2^ were recorded in the case of filtering the non-stabilized and stabilized oil emulsions, respectively, using BC membranes while the corresponding flux values, in the case of using CNF membrane, were 119 and 53 L/h/m^2^, respectively. The decrease in flux values over time, especially at the beginning, in the case of pure water flux is due to compaction of membranes by the action of the applied pressure, which decreases the porosity of the membranes [33]. In the case of filtering the oil emulsions, the further reduction of flux with time is due to the clogging of the pores of the membrane by the oil droplets and concentration polarization due to the accumulation of oil droplets on the filtration side. In addition, the filtered oil can form a gel-like layer at the surface of the membrane by the action of the applied pressure. It is noticed from the slope of the curves that the flux quickly reached a nearly steady state value in the case of CNF membrane than in the case of the BC membrane. In addition, it should be noted that the flux values mentioned are at the studied time and not the stable flux values, since some curves still show slopes at end of the test.

An important observation in case of BC is its higher wet strength, i.e., expected higher durability than the CNF membrane. This can allow cleaning of the BC membrane and its re-use while the CNF membrane is a single-use and disposable one.

The data of the flux curves above were analyzed using different mathematical model equations to identify the most probable reasons for decreasing the flux, i.e., fouling of the membranes. The following equations, which describe the possible reasons for decreasing the flux upon filtration at constant pressure were used [34]:

Complete pore blocking model: ln*J* = ln*J*_0_ − *K*_b_*t (3)

Standard pore blocking model: 1/(*J*^1/2^) = 1/(*J*_0_^1/2^) + *K*_s_*t (4)

Intermediate pore blocking model: 1/*J* = 1/*J*_0_ + *K*_i_*t (5)

Gel/cake filtration model: 1/*J*^2^ = 1/*J*_0_^2^ + *K*_c_*t (6)

where *J* is the permeation flux with time *t*, *J*_0_ is initial permeate flux and *K*_b_, *K*_s_, *K*_i_, and *K*_c_ are mass transfer coefficients for the corresponding filtration model equation.

According to these equations, the plots of ln(*J*) vs. *t*, (1/*J*^1/2^) vs. *t*, (1/*J*) vs. *t* and (1/*J*^2^) vs. *t* give curves with the slope equal to *K*_b_, *K*_s_, *K*_i_ and *K*_c_, and the intercept corresponds to ln(*J*_0_), (1/*J*_0_^1/2^), (1/*J*_0_) and (1/*J*_0_^2^), respectively. The coefficients of correlation (*R*^2^) obtained from the linear regression analysis of the curves are shown in Table 3 (curves are not shown). From the table, it is clear that the highest *R*^2^ values were obtained for the intermediate pore blocking and gel filtration models, i.e., these models are the best fitted to describe the decrease in flux upon filtration of oil emulsions (stabilized or non-stabilized) using BC and CNF membranes.

### 3.6. Removal Efficiency of Oil from Oil-in-Water Emulsions

**Figure 10:**
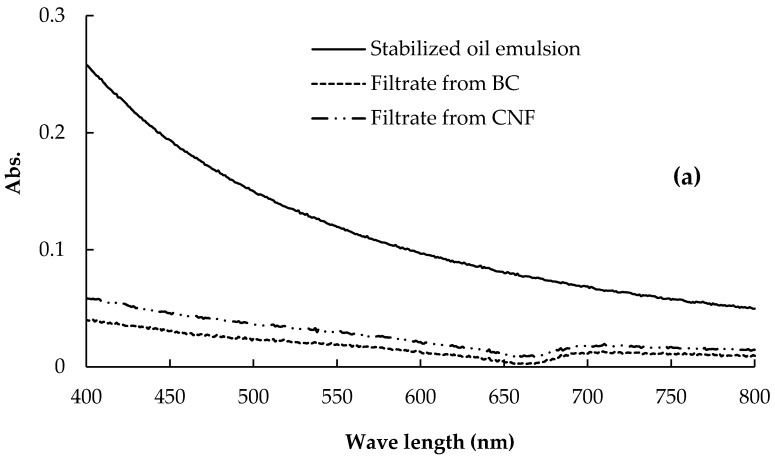

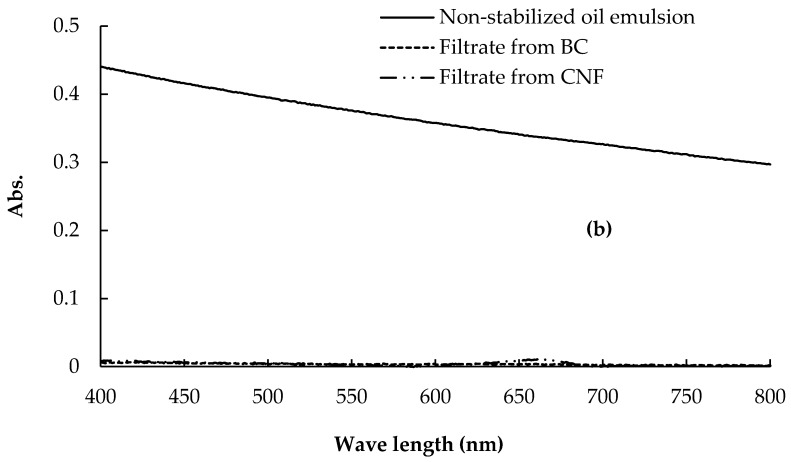
Visible spectra of: (**a**) stabilized and (**b**) non-stabilized oil emulsions before and after filtration through BC and CNF membranes.

The efficiency of CNF and BC membranes in the removal of oil from oil-in-water emulsion was estimated by following the visible light absorbance at 600 nm of the stabilized and non-stabilized oil emulsions before and after filtration through the membranes. BC membranes harvested after two days were used in the test since they showed the highest pure water flux than the other BC membranes. Figure 10 shows the visible spectra of stabilized and non-stabilized oil emulsion before and after filtration through the BC and NFC membranes. As shown in the figure, removal of oil from the stabilized oil emulsion by the different membranes was less than that in case of the non-stabilized one, probably due to the smaller particle diameter of the stabilized emulsion. In addition, aggregation of oil droplets during filtration of the non-stabilized emulsion may lead to easier separation. The efficiency of oil removal from the stabilized oil emulsion was 98.3% and 92.9% for BC and CNF membranes, respectively, while in the case of the non-stabilized oil emulsion the efficiency of removal was 99.3% and 97.9% for BC and CNF membranes, respectively. The higher efficiency of the BC membrane in removing oil from the stabilized oil emulsion could be due to its unique tight structure.

## 4. Conclusions

Never-dried BC and CNF membranes could be used for efficient removal of oil from non-stabilized and stabilized oil-in-water emulsions having droplet sizes of less than 1 µm. Increasing harvesting time of BC did not affect the porosity of the never-dried BC. However, due to its very thin structure, the two-day BC showed exceptional pure water flux as compared to BC harvested after longer times. Never-dried BC is more efficient in removal of oil from oil emulsions than the prepared CNF membrane with respect to flux and percentage of removal, especially in case of the stabilized oil-in-water emulsion. The higher flux values in the case of BC than those of CNF membranes was attributed mainly to that BC was never-dried, while CNF was dried at 105 °C after exchanging water with isopropyl alcohol, the conditions that resulted in a much more dense structure and less hydrophilic property. Fitting flux data of oil emulsions filtration to standard mathematical model equations showed that the decrease in flux noticed during filtration mainly occurs by intermediate blocking of the membrane’s pores and gel-like layer formation by oil droplets. The high wet strength of BC, its nanoporous structure and ability to remove sub-micron-sized contaminants make it a good candidate for environmentally-friendly ultrafiltration membranes.

## Figures and Tables

**Table 1:** Weight, thickness and water content of BC harvested after different incubation times.

Incubation Period (Days)	Wet Weight (g)	Dry Weight (g)	Water Content (%)	Wet Thickness (mm)	Dry Thickness (mm)
2	1.40	0.017	98.8	0.40	0.02
4	10.66	0.13	98.7	3.85	0.04
6	21.43	0.17	99.2	5.23	0.08
10	51.58	0.44	99.1	11.60	0.1

**Table 2:** Water flux values for filtering 300 mL of pure water by CNF membrane and BC membranes harvested after different times.

Sample	2-Day BC	4-Day BC	6-Day BC	10-Day BC	CNF
Water flux (L/h/m^2^)	845 ± 70	448 ± 45	441 ± 67	498 ± 52	192 ± 29

**Table 3:** Coefficient of correlation (*R*^2^) values for different fouling models for the filtration of oil–water emulsion by BC and CNF membranes.

Membrane	Coefficient of Correlation (*R*^2^)			
	Complete Blocking	Standard Pore Blocking	Intermediate Blocking	Gel/Cake Filtration
BC/non-stabilized oil emulsion	0.87	0.76	0.99	0.99
BC/stabilized oil emulsion	0.95	0.88	0.99	0.96
CNF/non-stabilized oil emulsion	0.88	0.82	0.95	0.99
CNF/stabilized oil emulsion	0.85	0.70	0.99	0.99

## Supplementary Materials

The following are available online at www.mdpi.com/2073-4360/9/9/388/s1.

## References

[1] Lee K.P., Arnot T.C., Mattia D. (2011). A review of reverse osmosis membrane materials for desalination-development to date and future potential. J. Membr. Sci..

[2] Zhao W., Moser C., Lindström M.E., Henriksson G., Li J. (2017). Cellulose nanofibers from softwood, hardwood and tunicate: preparation–structure–membrane performance interrelation. ACS Appl. Mater. Interfaces.

[3] Hassan M.L., Pandey J.K., Takagi H. (2015). Bagasse and rice straw nanocellulosic materials and their applications. Handbook of Polymer Nanocomposites. Processing, Performance and Application. Volume C: Polymer Nanocomposites of Cellulose Nanoparticles.

[4] Prasanth R., Nageswaran S., Thakur V.K., Ahn J.H., Thakur V.K. (2014). Electrospinning of cellulose: Process and applications. Nanocellulose Polymer Nanocomposites: Fundamentals and Applications.

[5] Xu F., Weng B., Materon L.A., Kuang A., Trujillo J.A., Lozano K. (2016). Fabrication of cellulose fine fiber based membranes embedded with silver nanoparticles via Forcespinning. J. Polym. Eng..

[6] Weng B., Xu F., Alcoutlabi M., Mao Y., Lozano K. (2015). Fibrous cellulose membrane mass produced via forcespinning^®^ for lithium-ion battery separators. Cellulose.

[7] Voisin H., Bergström L., Liu P., Mathew A.P. (2017). Nanocellulose-based materials for water purification. Nanomaterials.

[8] Kucińska-Lipka J., Gubanska I., Janik H. (2015). Bacterial cellulose in the field of wound healing and regenerative medicine of skin: Recent trends and future prospective. Polym. Bull..

[9] Jung H.I., Lee O.M., Jeong J.H., Jeon Y.D., Park K.H., Kim H.S., An W.G., Son H.J. (2010). Production and characterization of cellulose by *Acetobacter* sp. V6 using a cost-effective molasses-corn steep liquor medium. Appl. Biochem. Biotechnol..

[10] Hutchens S.A., Leon R.V., O’Neill H.M., Evans B.R. (2007). Statistical analysis of optimal culture conditions for *Gluconacetobacter hansenii* cellulose production. Lett. Appl. Microbiol..

[11] Wanichapichart P., Kaewnopparat S., Buaking K., Puthai W. (2002). Characterization of cellulose membranes produced by *Acetobacter xyllinum*. Songklanakarin J. Sci. Technol..

[12] Sai H., Fu R., Xing L., Xiang J., Li Z., Li F., Zhang T. (2015). Surface modification of bacterial cellulose aerogels’ web-like skeleton for oil/water separation. ACS Appl. Mater. Interfaces.

[13] Fang Q., Zhou X., Deng W., Zheng Z., Liu Z. (2016). Freestanding bacterial cellulose-graphene oxide composite membranes with high mechanical strength for selective ion permeation. Sci. Rep..

[14] Mautner A., Lee K.Y., Lahtinen P., Hakalahti M., Tammelin T., Li K., Bismarck A. (2014). Nanopapers for organic solvent nanofiltration. Chem. Commun..

[15] Takai M., Nonomura F., Inukai T., Fujiwara M., Hayashi J. (1991). Filtration and permeation characteristics of bacterial cellulose composite. Sen'i Gakkaishi.

[16] Paul U.C., Fragouli D., Bayer I.S., Athanassiou A. (2016). Functionalized cellulose networks for efficient oil removal from oil-water emulsions. Polymers.

[17] Kiss Z.L., Kertész S., Beszédes S., Hodúr C., László Z. (2013). Investigation of parameters affecting the ultrafiltration of oil-in-water emulsion wastewater. Desalin. Water Treat..

[18] Wandera D., Wickramasingh S.R., Husson S.M. (2012). Modification of ultrafiltration membranes with block copolymer nanolayers for produced water treatment: The roles of polymer chain density and polymerization time on performance. J. Membr. Sci..

[19] Ma H., Hsiao B.S., Chu B. (2011). Thin-film nanofibrous composite membranes containing cellulose or chitin barrier layers fabricated by ionic liquid. Polymer.

[20] Wandera D., Wickramasingh S.R., Husson S.M. (2011). Modification and characterization of ultrafiltration membranes for treatment of produced water. J. Membr. Sci..

[21] Ma H., Burger C., Hsiao B.S., Chu B. (2014). Fabrication and characterization of cellulose nanofiber based thin-membrane nanofibrous composite membranes. J. Membr. Sci..

[22] Hassan M.L., Bras J., Hassan E.A., Silard C., Mauret E. (2014). Enzyme-assisted isolation of cellulose nanofibersfrom date palm fruit stalks. Ind. Crops Prod..

[23] Wise L.E., Murphy M., D’Addieco A.A. (1946). Chlorite holocellulose, its fractionation and bearing on summative wood analysis and on studies on hemicelluloses. Pap. Trade J..

[24] Browning B.L. (1967). Methods of Wood Chemistry.

[25] Hassan M.L., Mathew A.P., Hassan E.A., El-Wakil N.A., Oksman K. (2012). Nanofibers from bagasse and rice straw: Process optimization and properties. Wood Sci. Technol..

[26] Czaja W., Romanovicz D., Brown R.M. (2004). Structural investigations of microbial cellulose produced in stationary and agitated culture. Cellulose.

[27] Sidiras D.K., Koullas D.P., Vgenopoulos A.G., Koukios E.G. (1990). Cellulose crystallinity as affected by various technical processes?. Cell. Chem. Technol..

[28] Cheng K.C., Catchmark J.M., Demirci A. (2009). Enhanced production of bacterial cellulose by using a biofilm reactor and its material property analysis. J. Biol. Eng..

[29] Lyu B., Wang H.D., Ma J.Z., Gao D.G., Jin P. (2016). Preparation and application of castor oil/nano-TiO_2_ composite fatliquoring agent via a Pickering emulsion method. J. Clean. Prod..

[30] Ding Z., Liu X., Zhang L. (2016). Enhancing the compatibility, hydrophilicity and mechanical properties of polysulfone ultrafiltration membranes with lignoellulose nanofibrils. Polymers.

[31] Albo J., Wang J., Tsuru T. (2014). Application of interfacially polymerized polyamide composite membranes to isopropanol dehydration: Effect of membrane pre-treatment and temperature. J. Membr. Sci..

[32] Albo J., Hagiwara H., Yanagishita H., Ito K., Tsuru T. (2014). Structural characterization of thin-film polyamide reverse osmosis membranes. Ind. Eng. Chem. Res..

[33] Mautner A., Lee K.Y., Tammelin T., Mathew A.P., Nedoma A.J., Li K., Bismarck A. (2015). Cellulose nanopapers as tight aqueous ultra-filtration membranes. React. Funct. Ploym..

[34] Peng H., Tremblay A.Y. (2008). Membrane regeneration and filtration modelling in treating oily wastewaters. J. Membr. Sci..

