# Supplementary material for: Use of Bacterial Cellulose and Crosslinked Cellulose Nanofibers Membranes for Removal of Oil from Oil-in-Water Emulsions
Source: Polymers (Basel). 2017 Aug 23;9(9):388. doi: 10.3390/polym9090388 (PMC6418680; doi:10.3390/polym9090388)
Supplement: Supplementary file 1 [file polymers-09-00388-s001.docx]

**(a)**

**(b)**

**Figure S1.** (**a**) Visible light spectra of oil emulsion of different concentrations and (**b**) standard curve for absorbance at 600 nm.


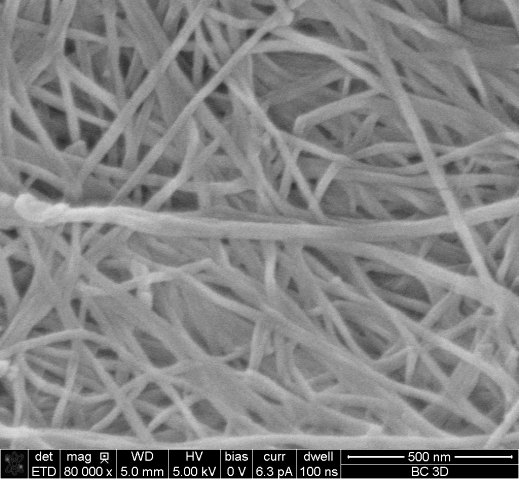

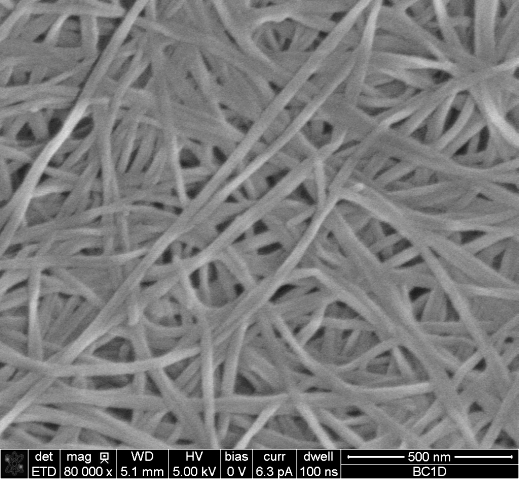


a

b


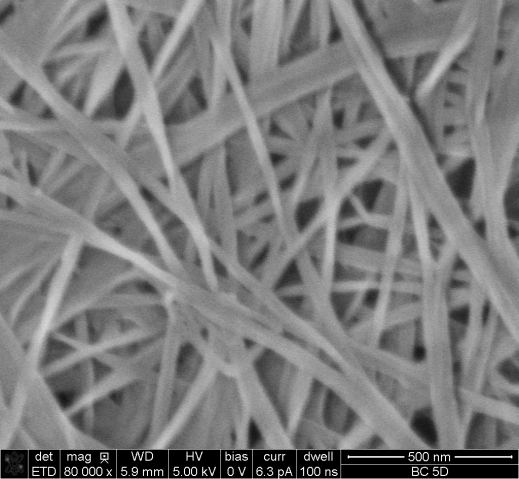

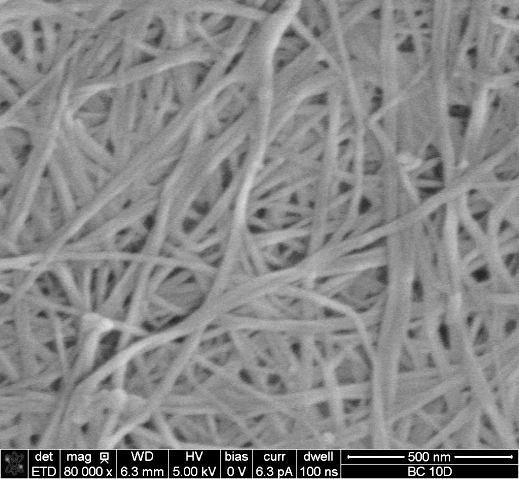


c

d

**Figure S2.** SEM images of BC membranes harvested at (**a**) 2, (**b**) 4, (**c**) 6, and (**d**) 10 days at 80000x.
